# Questioning the role of palmitoylethanolamide in psychosis: a systematic review of clinical and preclinical evidence

**DOI:** 10.3389/fpsyt.2023.1231710

**Published:** 2023-07-18

**Authors:** Riccardo Bortoletto, Fabiana Piscitelli, Anna Candolo, Sagnik Bhattacharyya, Matteo Balestrieri, Marco Colizzi

**Affiliations:** ^1^Unit of Psychiatry, Department of Medicine (DAME), University of Udine, Udine, Italy; ^2^Department of Chemical Sciences and Materials Technologies, Institute of Biomolecular Chemistry, National Research Council (CNR), Pozzuoli, Italy; ^3^Department of Psychosis Studies, Institute of Psychiatry, Psychology and Neuroscience, King's College London, London, United Kingdom

**Keywords:** Schizophrenia, bipolar disorder, major depressive disorder, antipsychotics, cannabidiol, nutraceuticals

## Abstract

**Introduction:**

The endocannabinoid (eCB) system disruption has been suggested to underpin the development of psychosis, fueling the search for novel, better-tolerated antipsychotic agents that target the eCB system. Among these, palmitoylethanolamide (PEA), an N-acylethanolamine (AE) with neuroprotective, anti-inflammatory, and analgesic properties, has drawn attention for its antipsychotic potential.

**Methods:**

This Preferred Reporting Items for Systematic Reviews and Meta-Analyses (PRISMA) 2020-compliant systematic review aimed at reappraising all clinical and preclinical studies investigating the biobehavioral role of PEA in psychosis.

**Results:**

Overall, 13 studies were eligible for data extraction (11 human, 2 animal). Observational studies investigating PEA tone in psychosis patients converged on the evidence for increased PEA plasma (6 human) and central nervous system (CNS; 1 human) levels, as a potential early compensatory response to illness and its severity, that seems to be lost in the longer-term (CNS; 1 human), opening to the possibility of exogenously supplementing it to sustain control of the disorder. Consistently, PEA oral supplementation reduced negative psychotic and manic symptoms among psychosis patients, with no serious adverse events (3 human). No PEA changes emerged in either preclinical psychosis model (2 animal) studied.

**Discussion:**

Evidence supports PEA signaling as a potential psychosis biomarker, also indicating a therapeutic role of its supplementation in the disorder.

**Systematic review registration:**

https://doi.org/10.17605/OSF.IO/AFMTK.

## 1. Introduction

Psychotic disorders—non-affective (e.g., schizophrenia (SCZ), schizophreniform disorder) and affective psychoses (e.g., bipolar disorder (BPAD), major depressive disorder with psychotic symptoms)—are a heterogeneous group of disabling mental health disturbances (1), sharing common phenomenological, neurobiological, and genetic characteristics (2–5). These conditions generally emerge between late adolescence and early adulthood (6), with a lifetime prevalence exceeding 3% (7–9), and severely affect the patients' and their families' quality of life. The dopaminergic and glutamatergic hypotheses still play a pivotal role in the attempt to describe the neurobiological mechanisms underlying psychosis (10–13), with potential for an integrated model explaining both positive (e.g., delusions, hallucinations) and negative (e.g., restricted emotional expression, avolition) psychotic symptoms (12).

To date, antipsychotic (AP) medications represent the cornerstone treatment for these conditions, although not always devoid of suboptimal clinical response and unpleasant side-effects (14, 15). Therefore, the exploration of other perturbed systems potentially underpinning psychotic disorders has aimed at identifying novel therapeutic targets. The endocannabinoid (eCB) system has been recognized as a mediator of the dopaminergic and glutamatergic systems via the cannabinoid receptor 1 (CB1) in the central nervous system (CNS), and found to be altered in the early phases of the disorder (16–19). Consistently, accumulating evidence has highlighted the therapeutic potential of the eCB system modulation. Particularly, cannabidiol (CBD) has shown promising results for both psychosis and clinical high-risk (CHR) for psychosis state (20–23). Further, reduced diversity of gut microbiota and gut-brain axis metabolic alterations associated have been indicated as having a putative role in the patho-etiological cascade toward psychosis (24). To this end, reduced microbiota diversity has been observed to contribute to common SCZ negative symptoms such as anhedonia and amotivation via eCB-like compound palmitoylethanolamide (PEA) fecal levels, warranting the possibility to target the gut microbiota-eCB axis (25). Finally, growing evidence emphasizes the importance of inflammation and oxidative stress in the stages preceding psychosis onset and throughout illness progression (26, 27).

PEA is an N-acylethanolamine (AE), produced “on demand” by different cell types as a response to actual or potential damage (28, 29). Importantly, PEA has been proven to down-regulate central and peripheral activity of mast cells and non-neuronal cells (e.g., astrocytes, microglia) (30–32) and to exert protective functions against glutamate neuro-toxicity, accounting for its naturally-occurring anti-inflammatory, analgesic, and anticonvulsant properties (33). It directly activates the Peroxisome Proliferator Activated Receptor-α (PPAR-α) and the GPR55, allosterically modulates the Transient Receptor Potential Vanilloid 1 (TRPV1), and indirectly interacts with CB1 and cannabinoid receptor 2 (CB2) (32, 34, 35). Due to the shared pharmacodynamic properties, PEA is considered as the endogenous equivalent of CBD (36, 37). A growing body of literature has confirmed the role of PEA in most neurobiological mechanisms underpinning several neuropsychiatric conditions both in clinical and preclinical settings (38–40).

### 1.1. Objectives

The effect of PEA over neuroinflammation and glutamate signaling may represent a promising biobehavioral mechanism underlying its clinical utility in psychosis. This systematic review aimed to collect and discuss all available clinical and preclinical data generated by studies investigating the role of PEA in non-affective and affective psychoses. We reviewed all interventional and observational studies, employing either retrospective or prospective methodological approaches with any PEA neuro-biological correlates investigated in psychosis.

## 2. Materials and methods

### 2.1. Inclusion and exclusion criteria

All clinical and preclinical evidence about the topic was gathered and systematically reviewed according to Preferred Reporting Items for Systematic Reviews and Meta-Analyses (PRISMA) 2020 guidelines (41). Inclusion criteria were defined as follows: 1. analytic, observational, and interventional studies; 2. studies assessing (i) acute or long-term effects of palmitoylethanolamide (PEA) administration over psychosis-related biological underpinnings (e.g., neuroimmune disruption, hypothalamus-pituitary-adrenal axis dysregulation); and behavioral features (e.g., negative psychotic symptoms, manic symptoms); or (ii) PEA and PEA signaling-related molecular marker (e.g., other endocannabinoids/acylethanolamines, PEA-related enzymes) modulation in peripheral tissues (e.g., plasma, serum), or in the central nervous system (e.g. cerebrospinal fluid, brain tissue) in psychosis and related conditions. Exclusion criteria were defined as outlined: 1. studies in which (i) PEA was not the intervention or outcome of interest (e.g., studies evaluating only exogenous cannabinoid administration or assessing endogenous cannabinoid levels); and (ii) PEA bio-behavioral correlates were not investigated with reference to psychosis; and (iii) PEA bio-behavioral correlates were not directly reported on; 2. reviews; 3. systematic reviews; 4. meta-analyses.

### 2.2. Search strategy and data extraction

A literature search was performed using electronic databases (PubMed, Scopus, and Web of Science) for any published original study written in English, on 16 January 2023. In order to be as much inclusive as possible, a combination of broad-meaning terms describing and/or concerning PEA (“palmitoylethanolamide,” “palmitylethanolamide,” “N-2-hydroxyethyl-hexadecanamide,” “N-2-hydroxyethyl-palmitate,” “N-palmitoylethanolamine,” “PEA,” and “palmitoyl-ethanolamine”) and psychosis (“schizophreni^*^,” ‘psychosis,” “psychoses,” “psychotic,” “bipolar,” “mania,” “manic”) was adopted. Reference lists of all selected studies were scrutinized to identify any adjunctive eligible evidence. Data screening and extraction were conducted according to a two-step selection process (conventional double-screening), performed by two researchers (RB and MC) independently from each other. In the instances of conflicting opinions regarding papers' inclusion, a consensus was sought through discussion with a third senior reviewer (MB).

### 2.3. Risk of bias assessment

In light of the methodological heterogeneity of collected evidence, quality of studies assessment was conducted in accordance to an adapted and suitably flexible set of criteria suggested by the Agency for Healthcare Research and Quality (AHRQ) guidance (42), in line with previous research in the field (38–40). Risk of systematic bias across human studies was ruled out by screening all papers for potential confounding factors (e.g., gender, age, smoking status, level of education). Furthermore, factors possibly accounting for similarities and differences between all studies were assessed, extracting information about study characteristics, including study design (e.g., observational, interventional), defined study population (for human studies: e.g., schizophrenia (SCZ) patients, clinical high-risk (CHR) subjects; for animal studies: e.g., mouse or rat model), age or developmental stage, gender, adequate psychosis model (for animal studies only: e.g., maternal deprivation, methylazoxymethanol acetate (MAM) prenatal exposure), PEA measure (e.g., PEA dosage and administration route, PEA assessment in tissues), adequate PEA evaluation (e.g., time of exposure, single or multiple assessments), defined control group, comparability of subjects (for human studies only), exclusion criteria/adjusting factors (for human studies only), statistical analyses, and declaration of fundings/sponsorship. The full study protocol is available at https://doi.org/10.17605/OSF.IO/AFMTK.

## 3. Results

### 3.1. Study selection

**Figure 1:**
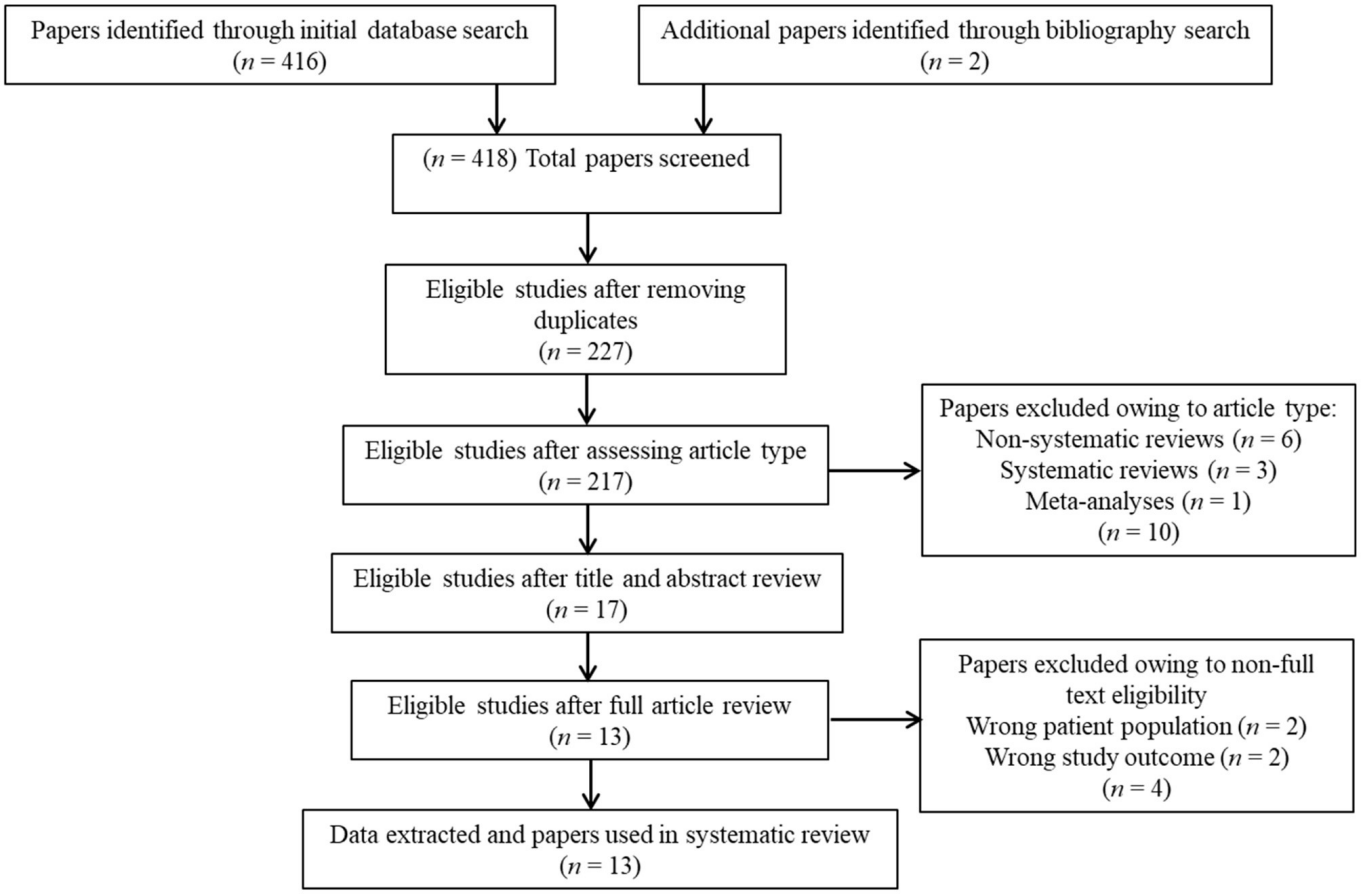
PRISMA flowchart of search strategy for systematic review.

Overall, 418 studies were retrieved through the initial data search. After removing duplicates as well as excluding articles owing to article type (e.g., non-systematic reviews, systematic reviews, meta-analyses), titles, abstracts, or full texts of all records were examined against the inclusion and exclusion criteria following a three-step screening process (Figure 1). A final list of thirteen studies was used for systematic analysis in this review, including 11 studies conducted only in human populations and two studies performed in animal models, exploring various aspects of palmitoylethanolamide (PEA) signaling pathway (Table 1). These include (i) *in vivo* PEA add-on treatment exposure in humans with different types of psychoses (e.g., non-affective psychosis, affective psychosis) or psychotic symptoms (e.g., hallucinations) (3 studies; Table 1); (ii) PEA quantitative blood assessment in humans with psychosis clinical high-risk (CHR) state (1 study; Table 1); (iii) PEA quantitative blood assessment in humans with different types of psychoses (e.g., non-affective psychosis, affective psychosis) at different stages of illness (5 studies; Table 1); (iv) PEA quantitative central nervous system (CNS; e.g., brain tissue, cerebrospinal fluid) assessment in humans with schizophrenia (SCZ; 2 studies; Table 1); (v) PEA quantitative brain tissue assessment in animal models of SCZ (2 studies; Table 1). Additional data on methodological quality of studies conducted in humans and animals are reported in Tables 2, 3. A brief synthesis of the main results is presented below and summarized in Table 1.

**Table 1:** Summary of clinical and preclinical studies investigating palmitoylethanolamide and its correlations to psychotic disorders.

**Study (year)**	**Country**	**Aim of study**	**Type of PEA study**	**Population**	**Total sample size**	**Outcome measure (test name or description)**	**Summary results**
Leweke et al. (43)	Germany	To assess PEA and other eCBs/AEs levels in SCZ patients	Quantitative assessment in humans	1. SCZ (*n*=10); 2. HC (*n*=11)	21	eCBs/AEs CSF levels (HPLC, GC/MS)	1. **PEA levels: SCZ** **>** **HC;** 2. **AEA levels: SCZ** **>** **HC**; 3. OEA levels: SCZ vs. HC, NS
Leweke et al. (44)	Germany	To assess PEA, other eCBs/AEs, and related enzymes levels in CBD-treated SCZ patients	Quantitative assessment in humans	1. CBD (*n*=20); 2. AMI (*n*=19)	39	eCBs/AEs and related enzymes serum levels (LC/MS, FAAH assay)	CBD group > AMI group: 1. **PEA levels: Day 14, Day 28** **>** **Baseline;** 2. **AEA levels: Day 14, Day 28** **>** **Baseline**; 3. **OEA levels: Day 14** **>** **Baseline;** Day 28 vs. Baseline, NS
Muguruza et al. (45)	Spain	To assess postmortem PEA and other eCBs/AEs levels in SCZ patients	Quantitative assessment in humans	1. AP-F (*n*=11); 2. AP-T (*n*=8); 3. CTRL (*n*=19)	38	eCBs/AEs brain tissue levels (LC/MS)	1. Effect on eCBs/AEs levels: (a) **2-AG levels: SCZ**, **↑**; **brain region**; SCZ x brain region interaction, NS; (b) **AEA levels: SCZ**, **↓**; brain region, NS; SCZ x brain region interaction, NS; (c) **DHEA levels: SCZ**, **↓**; **brain region**; **SCZ x brain region interaction**; (d) **PEA**, (e) **LEA levels: SCZ**, **↓**; **brain region**; SCZ x brain region interaction, NS; (f) **OEA levels**: SCZ, NS; **brain region**; SCZ x brain region interaction, NS; 2. **PEA brain tissue levels**: (a) **CB: SCZ** **<** **CTRL**; **AP-F** **<** **CTRL**; AP-T vs. CTRL, NS; AP-T vs. AP-F, NS; (b) HIP, (c) PFC: all comparisons, NS; 3. **LEA brain tissue levels**: (a) **CB: SCZ** **<** **CTRL**; other comparisons, NS; (b) HIP: all comparisons, NS; (c) **PFC: AP-T** **<** **CTRL**; other comparisons, NS; 4. **DHEA brain tissue levels:** (a) **CB: SCZ** **<** **CTRL**; **AP-F, AP-T** **<** **CTRL**; AP-F vs. AP-T, NS; (b) **HIP: SCZ** **<** **CTRL**; **AP-F** **<** **CTRL**; other comparisons, NS; (c) PFC: all comparisons, NS; 5. **AEA brain tissue levels**: (a) **CB: SCZ** **<** **CTRL**; **AP-F** **<** **CTRL**; other comparisons, NS; (b) **HIP: SCZ** **<** **CTRL**; **AP-F, AP-T** **<** **CTR**L; AP-F vs. AP-T, NS; (c) **PFC: AP-T** **<** **CTRL**; other comparisons, NS; 6. **2-AG brain tissue levels**: (a) CB: all comparisons, NS; (b) **HIP: SCZ** **>** **CTRL**; **AP-F** **>** **CTRL**; other comparisons, NS; (c) **PFC: SCZ** **>** **CTRL**; **AP-F** **>** **CTRL**; other comparisons, NS; 7. OEA brain tissue levels: all comparisons, NS; 8. **2-AG/PEA ratio**: (a) **CB**, (b) **PFC: SCZ** **>** **CTRL**; (c) HIP: SCZ vs. CTRL, NS; 9. **2-AG/other AEs ratio: SCZ** **>** **CTRL (all brain regions; all comparisons)**
Koethe et al. (46)	United States	To assess PEA and other eCBs/AEs levels in SCZ and BPAD discordant twin patients	Quantitative assessment in humans	1. SCZ discordant twin pairs: (a) SCZ (*n*=25); (b) noSCZ (*n*=25); 2. BPAD discordant twin pairs: (a) BPAD (*n*=7); (b) noBPAD (n=7); 3. HC twins (*n*=16)	80	eCBs/AEs plasma levels (LC/MS)	1. **PEA plasma levels: SCZ, noSCZ** **>** **HC**; SCZ vs. noSCZ, NS; **BPAD, noBPAD** **>** **HC** (NS after Bonferroni's correction); BPAD vs. noBPAD, NS; SCZ-transit vs. SCZ-non transit, NS; **SCZ discordant** **>** **BPAD discordant**; 2. **AEA plasma levels: SCZ, noSCZ** **>** **HC**; SCZ vs. noSCZ, NS; **BPAD, noBPAD** **>** **HC**; BPAD vs. noBPAD, NS; **SCZ-transit** **<** **SCZ-non transit**; 3. 2-AG plasma levels: SCZ vs. noSCZ vs. HC, NS; BPAD vs. noBPAD vs. HC, NS; **SCZ-transit** **<** **SCZ-non transit**; 4. OEA plasma levels: SCZ vs. noSCZ vs. HC, NS; BPAD vs. noBPAD vs. HC, NS; SCZ-transit vs. SCZ-non transit, NS
Appiah-Kusi et al. (47)	United Kingdom	To assess PEA and other eCBs/AEs levels in CT-exposed CHR patients	Quantitative assessment in humans	1. HC (*n*=58); 2. CHR (*n*=33)	91	eCBs/AEs plasma levels (LC/MS)	1. Group differences on AEs/eCBs **plasma levels**: (a) PEA: CHR > HC (trend effect); (b) **OEA**, (c) **AEA**, (d) **2-AG: CHR** **>** **HC**; 2. (a) **CT effect:** **↑PEA, AEA, 2-AG levels**; (b) **CHR effect:** **↑AEA, 2-AG levels**; (c) **CHR x CT interaction:** **↑PEA levels**; ↑ AEA levels (trend effect); 3. **Effects of 2 vs. 1 RF on AEs/eCBs plasma levels**: (a) **PEA**, (b) **AEA**, (c) **OEA**, (d) **2-AG: 2RF** **>** **1RF**; 4. **Effects of RF number on AEs/eCBs plasma levels**: (a) **PEA**, (b) **AEA**, (c) **OEA**, (d) **2-AG: noRF** **<** **1RF** **<** **2RF**; 5. **↑PEA levels:** **↑total CAARMS score**; **↑total CTQ score**; 6. ↑ AEA levels: ↑ total CAARMS score (trend effect)
Ibarra-Lecue et al. (48)	Spain	To assess PEA and other eCBs/AEs levels in DUAL patients	Quantitative assessment in humans	1. CUD (*n*=26); 2. HC1 (n=24); 3. SCZ (*n*=22); 4. HC2 (n=19); 5. DUAL (*n*=13); 6. HC3 (*n*=10)	114	1. eCBs/AEs plasma levels (HPLC/MS); 2. CB1R protein expression in PLTs (Western blot); 3. Inflammatory response measurements (ELISA)	1. CB1R protein expression: (a) **CUD main effect**; (b) **SCZ main effect**; (c) **CUD x SCZ interaction**; (d) **% from control: CUD, SCZ, DUAL** **<** **HC**; 2. eCBs/AEs plasma levels: (a) **SCZ main effect: PEA, OEA**; (b) **CUD main effect: PEA, AEA, DEA, LEA, NADA**; (c) **SCZ x CUD interaction: PEA, AEA, DEA, OEA**; (d) **PEA plasma levels (ng/ml): SCZ** **>** **DUAL; SCZ** **>** **HC** **>** **CUD**; other comparisons, NS; (e) **AEA plasma levels (ng/ml): SCZ** **>** **DUAL, CUD, HC**; other comparisons, NS; (f) **DEA plasma levels (ng/ml): SCZ** **>** **DUAL, CUD**; other comparisons, NS; (g) **OEA plasma levels (ng/ml): SCZ, CUD, HC** **>** **DUAL**; other comparisons, NS; (h) **NADA plasma levels (ng/ml): DUAL** **>** **HC**; other comparisons, NS; (i) 2-AG, (l) 1-AG, (m) LEA plasma levels (ng/ml): all comparisons, NS; 3. **IL-6 plasma levels (pg/ml): SCZ** **>** **DUAL, CUD, HC**; other comparisons, NS
Parksepp et al. (49)	Estonia	To assess PEA and other eCBs/AEs levels in FEP patients	Quantitative assessment in humans	1. FEP (*n*=54); 2. HC (*n*=58)	112	eCBs/AEs serum levels (HPLC/MS, flow injection analysis tandem MS)	1. eCBs/AEs serum levels: (a) PEA, (b) AEA, (c) LEA, (d) OEA: FEPb > HC (trend effect); FEP(0.6-year) vs. HC, NS; FEP(5.1-year) vs. HC, NS; (e) **2-AG**: FEPb < HC (trend effect); FEP(0.6-year) vs. HC, NS; **FEP(5.1-year)** **>** **HC**; 2. (eCBs/AEs)/2-AG ratio levels: (a) **PEA/2-AG: FEPb** **>** **HC**; FEP(0.6-year) vs. HC, NS; FEP(5.1-year) < HC (trend effect); (b) **AEA/2-AG**, (c) **OEA/2-AG: FEPb** **>** **HC**; FEP(0.6-year) vs. HC, NS; FEP(5.1-year) vs. HC, NS; (d) **LEA/2-AG: FEPb** **>** **HC**; FEP(0.6-year) vs. HC, NS; **FEP(5.1-year)** **<** **HC**; 3. (eCBs/AEs)/AEA ratio levels: (a) PEA/AEA, (b) OEA/AEA: FEPb vs. HC, NS; FEP(0.6-year) vs. HC, NS; FEP(5.1-year) vs. HC, NS; (c) **LEA/AEA**: FEPb vs. HC, NS; FEP(0.6-year) vs. HC, NS; **FEP(5.1-year)** **<** **HC**
Topuz et al. (50)	Turkey	To assess PEA and other eCBs/AEs levels in BPAD patients	Quantitative assessment in humans	1. Past depressive episode: (a) NoPastDEP (*n*=37); (b) PastDEP (*n*=42); 2. First episode type: (a) Manic/hypomanic (*n*=52); (b) Depressive (*n*=27)	79	eCBs/AEs serum levels (LC/MS)	1. eCBs/AEs serum levels: (a) AEA: NoPastDEP vs. PastDEP, NS; Manic/hypomanic vs. Depressive, NS; (b) **PEA: NoPastDEP** **<** **PastDEP**; **Manic/hypomanic** **<** **Depressive**; (c) **OEA: NoPastDEP** **<** **PastDEP**; Manic/hypomanic vs. Depressive, NS; (d) AEA: NoPastDEP vs. PastDEP, NS; Manic/hypomanic vs. Depressive, NS; 2. Correlations between illness course and eCBs/AEs serum levels: (a) **PEA:** **↑number of depressive episodes**, **↑**; **↑number of hypomanic episodes**, **↓**; **↑number of hospitalizations**, **↓**; **↑duration of VPA usage**, **↓**; other correlations, NS; (b) **AEA:** **↑duration of VPA usage**, **↑**; other correlations, NS; (c) **OEA:** **↑age of onset**, **↑;** **↑number of depressive episodes**, **↑**; other correlations, NS; (d) **2-AG:** **↑number of depressive episodes**, **↑**; other correlations, NS; 3. Relation of symptoms and eCBs/AEs serum levels: (a) **PEA: presence of depressive mood**, **↑**; **presence of increased sexual desire**, **↓**; **presence of anxiety**, **↑**; **presence of flight of ideas**, **↓**; **presence of delusion**, **↓**; **presence of grandiosity**, **↓**; presence of other symptoms, NS; (b) **AEA: presence of flight of ideas**, **↓**; **presence of increased motor activity**, **↓**; **presence of Schneiderian symptoms**, **↑**; presence of other symptoms, NS; (c) **OEA: presence of depressive mood**, **↑**; **presence of anxiety**, **↑**; presence of other symptoms, NS; (d) **2-AG: presence of euphoria**, **↑**; presence of other symptoms, NS; 4. Relation of medical history and eCBs/AEs serum levels: (a) **PEA: presence of another disease**, **↓**; any other medical history, NS; (b) AEA: all medical history, NS; (c) OEA: all medical history, NS; (d) **2-AG: presence of diabetes mellitus**, **↓**; **presence of another disease**, **↓**; any other medical history, NS
Brotini et al. (51)	Italy	To assess PEA add-on effects on psychotic symptoms in PD patients	*In vivo* treatment in humans	PD patients	30	nM-EDL assessment (MDS-UPDRS)	Effect on hallucinations and psychosis (nM-EDL scores): post-PEA vs. pre-PEA, NS
Salehi et al. (52)	Iran	To assess PEA add-on effects on negative symptoms in SCZ patients	*In vivo* treatment in humans	1. PEA (*n*=25); 2. PLB (*n*=25)	50	1. Symptoms assessment (PANSS, HDRS); 2. Adverse events assessment (ESRS, open-ended questions, comprehensive side effect checklist)	1. **Effect on PANSS negative: time**, **↓**; **time x treatment interaction**, **↓**; 2. **Effect on PANSS positive: time**, **↓**; time x treatment interaction, NS; 3. **Effect on PANSS general: time**, **↓**; **time x treatment interaction**, **↓**; 4. **Effect on PANSS total: time**, **↓; time x treatment interaction**, **↓**; 5. Effect on HDRS: time x treatment interaction, NS; 6. Effect on ESRS global score: time, NS; time x treatment interaction, NS; 7. Frequency of adverse events (drowsiness, dizziness, tremor, increased appetite, nervousness, restlessness, skin rash, blurred vision, fatigue, diarrhea, dry mouth, sore throat, tachycardia): PEA vs. PLB, NS
Abedini et al. (53)	Iran	To assess PEA add-on effects on acute mania in BPAD patients	*In vivo* treatment in humans	1. PEA (*n*=32); 2. PLB (*n*=31)	63	1. Symptoms assessment (YMRS, HDRS); 2. Adverse events assessment (ESRS, open-ended questions, comprehensive side effect checklist)	1. Effect on psychometric measures: (a) **YMRS: time x treatment interaction**; (b) ESRS: time x treatment interaction, NS; 2. **YMRS global score**: (a) Baseline, (b) Week 2, (c) Week 4: PEA vs. PLB, NS; (d) **Week 6: PEA** **<** **PLB**; 3. **YMRS score changes**: (a) From Baseline to Week 2: PEA vs. PLB, NS; (b) **From Baseline to Week 4**, (c) **From Baseline to Week 6: PEA** **>** **PLB**; 4. **HDRS global score**: (a) Baseline: PEA vs. PLB, NS; (b) **Week 6: PEA** **>** **PLB**; 5. HDRS score changes: From Baseline to Week 6: PEA vs. PLB, NS; 6. ESRS global score: (a) Baseline, (b) Week 1, (c) Week 2, (d) Week 4, (e) Week 6: PEA vs. PLB, NS; 7. ESRS score changes: all comparisons, NS; 8. Frequency of adverse events (drowsiness, dizziness, increased appetite, skin rash, diarrhea, dry mouth, sore throat, tachycardia): PEA vs. PLB, NS
Stark et al. (54)	Czech Republic/Italy	To assess PEA and other eCBs/AEs brain levels following CBD, CB1R antagonist/inverse agonist, and HAL in MAM rats	Quantitative assessment in animals	1. CTRL+VHI; 2. CTRL+CBD10; 3. CTRL+CBD30; 4. CTRL+AM251; 5. CTRL+HAL; 6. MAM+VHI; 7. MAM+CBD10; 8. MAM+CBD30; 9. MAM+AM251; 10. MAM+HAL	12-15 per group	eCBs/AEs brain levels (LC-APCI/MS)	1. Effects of peripubertal treatment (PND 19-39) on social interactions (SI test) from PND 100: (a) **effect on time: MAM; treatment; MAM x treatment interaction**; (b) **time: MAM+VHI** **<** **CTRL+VHI; MAM+CBD30** **>** **MAM+VHI; CTRL+AM251** **<** **CTRL+VHI; MAM+AM251** **>** **MAM+VHI; CTRL+VHI** **>** **CTRL+HAL**; other comparisons, NS; (c) effect on number of social interactions: MAM, NS; treatment, NS; MAM x treatment interaction, NS; (d) number of interactions: all comparisons, NS; 2. Effects of peripubertal treatment (PND 19-39) on exploratory activity (NORT, OFT) from PND 100: (a) **effect on DI: MAM**; treatment, NS; **MAM x treatment interaction**; (b) **DI: MAM+VHI** **<** **CTRL+VHI; MAM+CBD30** **>** **MAM+VHI**; other comparisons, NS; (c) effect on total exploration time: MAM, NS; treatment, NS; MAM x treatment interaction, NS; (d) total exploration time: all comparisons, NS; (e) effect on number of crossings: MAM, NS; treatment, NS; MAM x treatment interaction, NS; (f) number of crossings: all comparisons, NS; (g) effect on number of rearings: MAM, NS; treatment, NS; MAM x treatment interaction, NS; (h) number of crossings: all comparisons, NS; 3. Effects of peripubertal treatment (PND 19-39) on PFC CB1R expression from PND 100: (a) **effect on mRNA expression**: MAM, NS; treatment, NS; **MAM x treatment interaction**; (b) **% mRNA methylation: MAM+VHI** **<** **CTRL+VHI; MAM+CBD30** **>** **MAM+VHI; CTRL+AM251** **>** **CTRL+VHI; CTRL+HAL** **>** **CTRL+VHI; MAM+HAL** **>** **MAM+VHI**; other comparisons, NS; (c**) mRNA fold change: MAM+VHI** **>** **CTRL+VHI; MAM+CBD30** **<** **MAM+VHI; MAM+AM251** **<** **MAM+VHI**; other comparisons, NS; (d) **protein expression level: MAM+VHI** **>** **CTRL+VHI**; **CTRL+CBD30** **<** **CTRL+VHI; MAM+CBD30** **<** **MAM+VHI; MAM+AM251** **>** **CTRL+VHI; MAM+** **HAL** **>** **CTRL+** **VHI**; other comparisons, NS; 4. Effects of peripubertal treatment (PND 19-39) on PFC eCBs/AEs expression from PND 100: (a) effect on PEA levels: MAM, NS; treatment, NS; MAM x treatment interaction, NS; (b) PEA levels: all comparisons, NS; (c) **effect on 2-AG levels: MAM; treatment; MAM x treatment interaction**; (d) **2-AG levels: MAM+** **CBD30** **<** **MAM+** **VHI; CTRL+** **AM251** **>** **CTRL+** **VHI**; **MAM+** **AM251** **<** **MAM+** **VHI; CTRL+** **HAL** **>** **CTRL+** **VHI**; other comparisons, NS; (e) effect on AEA levels: MAM; treatment, NS; MAM x treatment interaction; (f) **AEA levels: CTRL+** **CBD30** **>** **CTRL+** **VHI**; other comparisons, NS; (g) effect on OEA levels: MAM, NS; treatment, NS; MAM x treatment interaction, NS; (b) OEA levels: all comparisons, NS
Di Bartolomeo et al. (55)	Czech Republic/Italy	To assess PEA and other eCBs/AEs brain levels following CBD in pTHC rats	Quantitative assessment in animals	1. CTRL+VHI; 2. CTRL+CBD; 3. pTHC+VHI; 4. pTHC+CBD	3-20 per group	eCBs/AEs brain levels (LC/MS)	1. Effects of pTHC on neonatal behavior: (a) **righting (PND 1-2)**, (b) **cliff aversion (PND 2-8)**, (c) **forelimb placing (PND 3-9)**, (d) **forelimb grasping (PND 2-4)**, (e) **bar holding (PND 5)**, (f) **negative geotaxis (PND 3-4)**, (g) **nest time**: **pTHC+VHI** **<** **CTRL+VHI**; (h) nest exploration: pTHC+VHI vs. CTRL+VHI, NS 2. Effects of pTHC on PFC eCBs/AEs expression at PND 10: (a) PEA, (b) AEA, (c) OEA levels: pTHC+VHI vs. CTRL+VHI, NS; (d) **2-AG levels: pTHC+VHI** **<** **CTRL+VHI**; (e) **Magl**, (f) **Faah mRNA expression: pTHC+VHI** **>** **CTRL+VHI**; (g) Cnr1, (h) Trpv1, (i) other eCBs/AEs enzymes mRNA expression: pTHC+VHI vs. CTRL+VHI, NS; 3. Effects of pTHC on PFC Drd2 gene expression at PND 10: pTHC+VHI > CTRL+VHI (trend effect); 4. Effects of peripubertal CBD (PND 19-39) on adult behavior: (a) number of crossings, (b) number of rearings (OFT): all comparisons, NS; (c) **SI time: pTHC+VHI** **<** **CTRL+VHI**; **pTHC+CBD** **<** **pTHC+VHI**; (d) SI number of interactions: all comparisons, NS; (e) **discrimination index (NORT): pTHC+VHI** **<** **CTRL+VHI**; **pTHC+CBD** **>** **pTHC+VHI**; (f) time (NORT): all comparisons, NS; 5. Effects of peripubertal CBD (PND 19-39) on PFC genes expression from PND 100: (a) %DNA methylation Cnr1 gene: all comparisons, NS; (b) **%DNA methylation Drd2 gene: pTHC+VHI** **<** **CTRL+VHI**; **CTRL+CBD** **<** **CTRL+VHI**; **pTHC+CBD** **>** **pTHC+VHI**; other comparisons, NS; (c) **Cnr1 relative gene expression: pTHC+VHI** **>** **CTRL+VHI**; other comparisons, NS; (d) **Drd2 relative gene expression: pTHC+VHI** **>** **CTRL+VHI; pTHC+CBD** **>** **CTRL+CBD**; (e) Cnr1 protein expression level: all comparisons, NS; (f) **Drd2 protein expression level: pTHC+VHI** **>** **CTRL+VHI**; other comparisons, NS; 6. Effects of peripubertal CBD (PND 19-39) on PFC eCBs/AEs expression from PND 100: (a) PEA levels: CTRL+VHI vs. pTHC+VHI vs. pTHC+CBD, NS; (b) **2-AG levels: pTHC+VHI** **<** **CTRL+** **VHI**; **pTHC+** **CBD** **<** **CTRL+** **VHI**; pTHC+CBD vs. pTHC+VHI, NS; (c) **AEA levels: pTHC+** **VHI** **>** **CTRL+** **VHI**; pTHC+VHI vs. pTHC+CBD, NS; pTHC+CBD vs. CTRL+VHI, NS; (d) OEA levels: all comparisons, NS

< , Lower/less than; >, Greater/more than; ↑, Increase; ↓, Decrease; 2-AG, 2-Arachidonoylglycerol; AEA, Anandamide; AEs, Acylethanolamines; AM251, CB1 antagonist/inverse agonist; AMI, Amisulpride; AP-F, Antipsychotic-free patients; AP-T, Antipsychotic-treated patients; BPAD, Bipolar Affective Disorder; CAARMS, Comprehensive Assessment of At-Risk Mental State; CB, Cerebellum; CB1R, Cannabinoid receptor type 1; CBD, Cannabidiol; CBD10, Cannabidiol (10 mg/kg/day); CBD30, Cannabidiol (30 mg/kg/day); CHR, Clinical High-Risk; Cnr1, Cannabinoid CB1 receptor gene; CSF, Cerebrospinal fluid; CT, Childhood trauma; CTQ, Childhood Trauma Questionnaire; CTRL, Control group; CUD, Cannabis Use Disorder; DEA, Docosatetraenylethanolamide; DHEA, N-docosahexaenoylethanolamine; DI, Discrimination index; DNA, Deoxyribonucleic acid; Drd2, Dopamine D2 receptor gene; DUAL, Dual diagnosis; eCBs, Endocannabinoids; ELISA, Enzyme linked immunosorbent assay; ESRS, Extrapyramidal Symptom Rating Scale; FAAH/Faah, Fatty Acid Amide Hydrolase; FEP, First-episode psychosis; FEPb, First-episode psychosis (baseline); GC/MS, Gas Chromatography-Mass Spectrometry; HAL, Haloperidol; HC, Healthy controls; HDRS, Hamilton Depression Rating Scale; HIP, Hippocampus; HPLC, High Pressure Liquid Chromatography; HPLC/MS, High Pressure Liquid Chromatography-Mass Spectrometry; IL-6, Interleukin 6; LC/MS, Liquid Chromatography-Mass Spectrometry; LC-APCI/MS, Liquid Chromatography-Atmospheric Pressure Chemical Ionization-Mass Spectrometry; LEA, Dihomo-linolenoylethanolamine; Magl, Monoacylglycerol lipase; MAM, Methylazoxymethanol acetate; MDS-UPDRS, MDS-Unified Parkinson's Disease Rating Scale; mRNA, Messenger ribonucleic acid; n, Sub-sample size; NADA, N-Arachidonoyldopamine; ng/ml, Nanograms per milliliter; nM-EDL, Non-Motor Aspects of Experiences of Daily Living; NoPastDEP, No prior depressive episodes; NORT, Novel object recognition test; noBPAD, healthy twins of BPAD patients; noSCZ, healthy twins of SCZ patients; NS, Not significant; OEA, Oleoylethanolamide; OFT, Open-field test; PANSS, Positive and Negative Syndrome Scale; PastDEP, At least one prior depressive episode; PD, Parkinson's Disease; PEA, Palmitoylethanolamide; PFC, Prefrontal cortex; PLB, Placebo; PLTs, Platelets; PND, Postnatal day; pTHC, Perinatal THC; RF, Risk factor; SCZ, Schizophrenia; SI, Social interaction; THC, Delta-9-tetrahydrocannabinol; Trpv1, Transient receptor potential vanilloid 1 gene; VHI, Vehicle; VPA, Valproic Acid; vs., Compared to; YMRS, Young Mania Rating Scale. Bold font emphasizes statistically significant results.

**Table 2:** Methodological quality of clinical studies investigating palmitoylethanolamide and its correlations to psychotic disorders.

**Study (year)**	**Study design**	**Defined study population**	**Age (years, mean ± SD)**	**Male gender count (%)**	**PEA measure**	**Adequate PEAEvaluation**	**Control**	**Comparability of Subjects**	**Excluded/adjusted for confounding factors**	**Statistical analyses**	**Funding or sponsorship**
Leweke et al. (43)	√ Analytic, observational	√ SCZ or schizophreniform disorder patients: DSM-IV; BPRS	√/X SCZ: 27.7 ± 9.6	√/X SCZ: 7 (70%)	√ CSF levels	√ Single assessment	√ HC	√ Matched for age	X	√*t*-test	√
Leweke et al. (44)	√ Analytic, observational	√ SCZ or schizophreniform disorder patients: DSM-IV; 18-50 years; BPRS ≥ 36; BPRS THOT factor ≥ 12	√ CBD: 29.7 ± 8.3; AMI: 30.6 ± 9.4	√ CBD: 15 (75%); AMI: 17 (89%)	√ Serum levels	√ Multiple assessment (baseline, day 14, day 28)	√ AMI	√/X Matched for age, weight, pulse, blood pressure, gender, PANSS, BPRS, SAS, EPS; not matched for CGI severity, Lorazepam mg/day	√ Excluded if positive UDS, history of SUDs, previous depot antipsychotic treatment (< 3 months before the study), history of treatment resistance, present relevant/unstable condition, pregnancy, or breastfeeding	√ Mixed effects repeated measures model (unstructured covariance matrix), Fisher's exact test	√
Muguruza et al. (45)	√ Analytic, observational	√ Postmortem SCZ patients' brain samples: DSM-IV	√ AP-F: 45 ± 4; AP-T: 49 ± 5; CTRL: 45 ± 3	√ AP-F: 9 (81.8%); AP-T: 6 (75%); CTRL: 15 (78.9%)	√ Brain tissue levels	√ Single assessment	√ CTRL	√ Matched for age, gender, PMI, pH, RIN, storage (months)	√ Excluded if positive toxicological test for cannabis; HC excluded if history of neuropsychiatric disorder, history of drug abuse	√ Two-way ANOVA, Fisher LSD test, Pearson's coefficient, *t*-test, one-way ANOVA, Dunnett's multiple comparison *post-hoc* test	√
Koethe et al. (46)	√ Analytic, observational	√ SCZ or BPAD discordant twin patients: DSM-III-R; stable clinical condition; SANS; clinical records review; GAF scale	√ SCZ discordant: 30; BPAD discordant: 33; HC twins: 31	√ SCZ discordant: 14 (56%); BPAD discordant: 1 (14.29%); HC twins: 3 (27.27%)	√ Plasma levels	√ Single assessment	√ noSCZ; noBPAD; HC	X	√ Excluded if positive UDS	√ Wilcoxon rank sum test, exact Wilcoxon signed rank test, Bonferroni's correction	√
Appiah-Kusi et al. (47)	√ Analytic, observational	√ CHR individuals: CAARMS criteria	√ HC: 25.05 ± 4.90; CHR: 23.82 ± 5.28	√ 1. HC: 53.00%; 2. CHR: 51.00%	√ Plasma levels	√ Single assessment	√ HC	√ Matched for age, gender, current CU	√ Excluded if history of psychotic or manic episode, past or current CNS disorder, current substance dependence (DSM-IV), IQ < 70, any contraindications to CBD treatment or MRI, drug use during the entire study	√ ANCOVA, *t*-test, chi-square, correlation analysis	√
Ibarra-Lecue et al. (48)	√ Analytic, observational	√ SCZ, CUD, or DUAL patients: SCID (DSM-IV, DSM-IV-TR); ICD	√ CUD: 32.5 ± 1.9; HC1: 32.8 ± 2.0; SCZ: 48.9 ± 1.8; HC2: 49 ± 2.1; DUAL: 38.0 ± 2.9; HC3: 37.3 ± 3.4;	√ CUD: 21 (80.77%); HC1: 19 (79.17%); SCZ: 13 (59.09%); HC2: 10 (52.63%); DUAL: 12 (92.31%); HC3: 10 (100%)	√ Plasma levels	√ Single assessment	√ HC1; HC2; HC3	√ Matched for age, gender	√ HC excluded if any neuropsychiatric disease, any past 2 years CU	√ Two-way ANOVA, Tukey's test	√
Parksepp et al. (49)	√ Analytic, observational	√ FEP patients: ICD-10; DUP < 3 years; no AP use before the study; 18–45 years	√ FEPb: 26.6 ± 6.1; FEP(0.6-year): 27.3 ± 6.4; FEP(5.1-year): 31.8 ± 5.9; HC: 24.7 ± 4.5	√ FEPb: 31 (57%); FEP(0.6-year): 27 (51%); FEP(5.1-year): 23 (43%); HC: 24 (44%)	√ Serum levels	√ Multiple assessment (baseline, 0.59 ± 0.06 years after baseline, 5.15 ± 1.25 years after baseline)	√ HC	√/X FEP and HC matched for age, gender, smoking status, BMI; FEP and HC not matched for length of education; FEP groups matched for AP dose; FEP groups not matched for BMI, BPRS score	√ Excluded if current organic or drug-induced psychosis, current psychotic disorders due to other medical conditions; Adjusted for gender, age at first visit, smoking status, Δt between the visits	√ Shapiro-Wilk test, *t*-test, repeated measure ANOVA, Scheffé *post-hoc* test, chi-square test, LME models, maximum likelihood method, likelihood ratio test, FDR procedure	√
Topuz et al. (50)	√ Analytic, observational	√ BPAD patients: DSM-5; euthymic period; 18–65 years	√ 42.40 ± 1.10	√ 35 (44.30%)	√ Serum levels	√ Single assessment	X	NA	X	√ Shapiro-Wilk test, *t*-test, Mann-Whitney U test, Spearman correlation analysis	√
Brotini et al. (51)	√ Analytic, observational, interventional	√ levodopa treated PD patients (PDSBB criteria): (a) HY scale > 0; (b) MMSE ≥26/30; (c) age>18 years; (d) levodopa therapy (eventually other PD medication) without modification over four consecutive weeks	√ 73 ± 8	√ 14 (46.67%)	√ um-PEA 600 mg	√ Twice per day administration (12 weeks), then daily administration (36 weeks)	X	NA	√ Excluded if other forms of parkinsonism, other forms of dementia, unreliable patients, non-compliant patients	√ GLMM, Wilcoxon signed-rank test, Bonferroni's correction, Tukey-Kramer adjusted test	√
Salehi et al. (52)	√ Analytic, observational, interventional	√ SCZ patients: 18–60 years; SCID (DSM-5); illness duration ≥ 2 years; PANSS negative ≥15; HDRS < 14; clinical stability on stable risperidone (PANSS total change ≤ 20% on 2 subsequent assessments within 2 weeks)	√ PEA: 33.76 ± 6.93; PLB: 36.80 ± 9.60	√ 1. PEA: 23 (92%); 2. PLB: 21 (84%)	√ PEA 600 mg (oral administration)	√ Twice per day administration (8 weeks)	√ PLB	√ Matched for age, gender, literacy, smoking status, marital status, overall SCZ duration, baseline HDRS, baseline PANSS, baseline ESRS	√ Excluded if IQ < 70, history of head trauma, prior 3 months history of ECT, prior 6 months substance or alcohol dependence, breastfeeding, pregnancy, suicidal ideation, history of neurosurgery, current acute or chronic medical disease, history of allergy to risperidone or PEA	√ Shapiro-Wilk test, Q-Q probability graphics, *t*-test, Levene's test, Fisher's exact test, ANOVA, Greenhouse-Geisser test	√
Abedini et al. (53)	√ Analytic, observational, interventional	√ BPAD patients: SCID (DSM-5); MINI; moderate to severe manic episode assessed (YMRS)	√ PEA: 30.78 ± 9.80; PLB: 32.74 ± 9.04	√ 1. PEA: 21 (71.9%); 2. PLB: 20 (64.5%)	√ PEA 600 mg (oral administration)	√ Twice per day administration (6 weeks)	√ PLB	√ Matched for age, gender, education, smoking status, marital status, overall BPAD duration, baseline HDRS, baseline YMRS, baseline ESRS	√ Excluded if IQ < 70, history of allergy to lithium, risperidone, or PEA, substance dependence (except nicotine and caffeine), receiving manic-inducing medications, metabolic disorders such as hypothyroidism or hyperthyroidism, current severe hepatic disease	√*t*-test, chi-square, Fisher's exact test, general linear model repeated-measures analysis, Mauchly test, Greenhouse-Geisser test	√

< , Lower/less than; >, Greater/more than; ≤ , Less than or equal to; ≥, More than or equal to; AMI, Amisulpride; ANCOVA, Analysis of Covariance; ANOVA, Analysis of Variance; AP, Antipsychotic; AP-F, Antipsychotic-free patients; AP-T, Antipsychotic-treated patients; BMI, Body Mass Index; BPAD, Bipolar Affective Disorder; BPRS, Brief Psychiatric Rating Scale; CAARMS, Comprehensive Assessment of At-Risk Mental State; CBD, Cannabidiol; CGI, Clinical Global Impression; CHR, Clinical High-Risk; CNS, Central nervous system; CSF, Cerebrospinal fluid; CTRL, Control group; CU, Cannabis use; CUD, Cannabis Use Disorder; DSM-5, Diagnostic and Statistical Manual of mental disorders, Fifth Edition; DSM-III-R, Diagnostic and Statistical Manual of mental disorders, Third Edition Revised; DSM-IV, Diagnostic and Statistical Manual of mental disorders, Fourth Edition; DSM-IV-TR, Diagnostic and Statistical Manual of mental disorders, Fourth Edition-Text Revision; DUAL, Dual diagnosis; DUP, Duration of untreated psychosis; ECT, Electroconvulsive therapy; EPS, Extrapyramidal Symptoms; ESRS, Extrapyramidal Symptom Rating Scale; FDR, False discovery rate; FEP, First-episode psychosis; FEPb, First-episode psychosis (baseline); GAF, Global Assessment of Functioning; GLMM, Generalized Linear Mixed Model; HC, Healthy controls; HDRS, Hamilton Depression Rating Scale; HY, Hoehn and Yahr; ICD, International Classification of Diseases; ICD-10, International Classification of Diseases, Tenth Edition; IQ, Intelligence Quotient; LME, Linear mixed-effects; LSD, Least Significant Difference; mg, Milligrams; mg/day, Milligrams per day; MINI, Mini-International Neuropsychiatric Interview; MMSE, Mini Mental State Examination; MRI, Magnetic Resonance imaging; NA, Not applicable; noBPAD, healthy twins of BPAD patients; noSCZ, healthy twins of SCZ patients; PANSS, Positive and Negative Syndrome Scale; PD, Parkinson's Disease; PDSBB, PD Society Brain Bank; PEA, Palmitoylethanolamide; PLB, Placebo; PMI, Postmortem interval; RIN, RNA intergrity number; SANS, Scale for the Assessment of Negative Symptoms; SAS, Social Anxiety Scale; SCID, Structured Clinical Interview for DSM; SCZ, Schizophrenia; SD, Standard Deviation; SUDs, Substance Use Disorders; THOT, Thought Disorder; UDS, Urine drugs screening; um-PEA, Ultramicronized-PEA; YMRS, Young Mania Rating Scale; Δt, Time period.

**Table 3:** Methodological quality of preclinical studies investigating palmitoylethanolamide and its correlations to psychotic disorders.

**Study (year)**	**Study design**	**Defined study population**	**Adequate psychosis model**	**Developmental stage**	**Gender**	**PEA Measure**	**Adequate PEA evaluation**	**Control**	**Statistical analyses**	**Funding or sponsorship**
Stark et al. (54)	√ Analytic, observational	√ Offsprings of MAM-exposed Sprague-Dawley rats	√ MAM 22 mg/Kg ip administration on GD 17	√ from PND 100	√ Male	√ Brain tissue levels	√ Single assessment	√ CTRL+VHI; CTRL+CBD10; CTRL+CBD30; CTRL+AM251; CTRL+HAL	√ Shapiro-Wilk test, two-way ANOVA, Fisher's LSD, *t*-test	√
Di Bartolomeo et al. (55)	√ Analytic, observational	√ Offsprings of THC-exposed Sprague-Dawley rats	√ THC 5 mg/Kg oral administration from GD 15 to PND 9	√ PND 10; from PND 100	√ Male	√ Brain tissue levels	√ Double assessment	√ CTRL+VHI; CTRL+CBD	√ two-way ANOVA, Bonferroni's correction, Mann-Whitney U test, *t*-test, Shapiro-Wilk test	√

AM251, CB1 antagonist/inverse agonist; ANOVA, Analysis of Variance; CBD, Cannabidiol; CBD10, Cannabidiol (10 mg/kg/day); CBD30, Cannabidiol (30 mg/kg/day); CTRL, Control group; GD, Gestational day; HAL, Haloperidol; ip, intra-peritoneal; LSD, Least Significant Difference; MAM, Methylazoxymethanol acetate; mg/Kg, Milligrams per kilogram; PEA, Palmitoylethanolamide; PND, Postnatal day; THC, Delta-9-tetrahydrocannabinol; VHI, Vehicle.

### 3.2. *In vivo* palmitoylethanolamide (PEA) add-on treatment exposure in humans with different types of psychoses or psychotic symptoms

Three human studies have addressed this area (Table 1) using similar but not overlapping methodologies in terms of study population [chronic schizophrenia (SCZ) patients (52), bipolar disorder (BPAD) patients with manic symptoms (53), Parkinson's disease (PD) patients (51)], PEA formulation [oral native PEA (52, 53), oral Ultramicronized (um)-PEA (51)], PEA dosage [600 milligrams (mg) daily (51), 600 mg twice/daily (51–53)], and PEA period of exposure [6 weeks (53), 8 weeks (52), 12 months (51)]. Apart from a single study lacking a controlled condition (51), all studies adopted a randomized, double-blind, placebo-controlled design (52, 53). Overall, results indicated a beneficial effect of PEA adjunctive therapy on residual negative and general psychopathological symptoms, but not positive symptoms, of risperidone-treated SCZ patients (52), as well as on manic symptoms of lithium- plus risperidone-treated BPAD patients (53). Coherent data emerged regarding depressive symptomatology, which did not appear to be improved in both SCZ and BPAD patients treated with PEA add-on as compared to placebo (52, 53). A single study addressing the effect of PEA over non-motor symptoms among levodopa-treated PD patients, showed no reduction in the number of subjects presenting with hallucinations and psychosis (51). Noteworthy, PEA was well-tolerated, in the absence of extrapyramidal symptoms or any other relevant side effect across the three studies, and for the entire duration of the compound administration.

### 3.3. Palmitoylethanolamide quantitative blood assessment in humans with psychosis clinical high-risk state

This systematic reappraisal identified a single human study specifically assessing peripheral blood PEA levels in individuals suffering from CHR state, as compared to healthy controls (HC) (47) (Table 1). PEA levels tended to be elevated in CHR patients, even though just approaching statistical significance. Intriguingly, PEA levels appeared to be significantly higher in those who were both CHR and had been exposed to childhood trauma (CT), compared to individuals having none of the above-mentioned risk factors or one risk factor alone. Furthermore, a significant positive correlation between PEA levels and the severity of CHR state and CT was observed (47).

### 3.4. Palmitoylethanolamide quantitative blood assessment in humans with different types of psychoses at different stages of illness

Most of the studies retrieved investigated peripheral blood PEA levels in patients with non-affective [e.g., schizophrenia (SCZ) (44, 46, 48, 49) and schizophreniform psychosis (44)] or affective [e.g., bipolar disorder (BPAD) (46, 50)] psychoses at different stages of illness (Table 1). Two studies converged on the evidence of higher PEA levels in SCZ patients than in healthy controls (HC) (46, 48), one of which further suggesting significantly higher PEA plasma concentration in SCZ patients compared to patients meeting criteria for cannabis use disorder (CUD) or dual diagnosis of CUD and SCZ (48). Interestingly, compared to HC, unaffected twin siblings of SCZ patients also showed increased PEA levels, that did not differ from those of patients (46). Remarkably, antipsychotic (AP)-naïve first-episode psychosis (FEP) patients compared to HC showed a tendency to elevated PEA plasma levels and a significantly higher PEA/2-arachidonoylglycerol (2-AG) ratio, with both that subsided an average of 0.6 and 5.1 years after the initiation of AP treatment (49). The modulating effect of AP treatment over acylethanolamines (AEs) levels was also investigated through a double-blind, randomized, parallel-group, controlled clinical trial, showing elevated serum PEA concentration in cannabidiol (CBD)-treated SCZ patients, compared with those treated with the antipsychotic amisulpride (44). Studies measuring PEA levels among BPAD patients showed a less pronounced increase in affected and unaffected siblings of illness-discordant twin couples, when compared to HC (46). Further, a higher PEA plasma concentration was found in BPAD patients having first episode as depression than in those who had their first episode as mania (50) as well as in those who had at least one depressive episode than in patients who had no prior depressive episodes (50). Finally, PEA levels were increased according to the number of depressive episodes and the presence of depressive mood and anxiety, while inversely correlating with the number of hypomanic episodes, the number of hospitalizations, the duration of valproate (VPA) treatment, sexual desire, presence of flight of ideas, delusion, and grandiosity (50).

### 3.5. Palmitoylethanolamide quantitative central nervous system assessment in humans with schizophrenia

Two studies analyzed PEA levels in the CNS of patients with psychosis (43, 45) (Table 1). In particular, PEA levels were reported to be elevated in the cerebrospinal fluid (CSF) of SCZ and schizophreniform psychosis patients compared with healthy controls (HC) (43). Conversely, a study on postmortem brain samples from subjects diagnosed with SCZ compared to controls indicated lower PEA levels in the cerebellum of antipsychotic (AP)-free patients only (45). No significant differences in PEA brain quantification were detected among all study groups in the other brain areas investigated (45).

### 3.6. Palmitoylethanolamide quantitative brain tissue assessment in animal models of schizophrenia

In total, two studies evaluated PEA levels in the prefrontal cortex (PFC) (54, 55), hippocampus (HIP) (54), and nucleus accumbens (NAc) (54) of rats exposed to either prenatal methylazoxymethanol acetate (MAM) (54) or perinatal delta-9-tetrahydrocannabinol (THC) (55), which present with many SCZ-relevant biobehavioral deficits at neonatal age (55) and adulthood (54, 55) (Table 1). While modulating endocannabinoids [eCBs; e.g., anandamide (AEA) and 2-acylglycerol (2-AG) (54, 55)] and other acylethanolamines [AEs; e.g., oleoylethanolamide (OEA) (54)], both MAM and THC exposure did not significantly affect PEA levels in all investigated brain areas, as neither did the peripubertal exposure to cannabidiol (CBD) (54, 55), Cannabinoid receptor type 1 (CB1)-antagonist/agonist AM251 (54), and first-generation antipsychotic haloperidol (HAL) (54), compared to control conditions.

## 4. Discussion

This is the first systematic review of all evidence exploring the biobehavioral correlates of palmitoylethanolamide (PEA) in psychosis across human and animal studies. Unlike previous research in this field (38–40), the greatest majority of records included consisted of human studies. Existing reviews focusing on the role of the major phytocannabinoid cannabidiol (CBD) as a potential treatment for schizophrenia (SCZ) patients (20, 56, 57) gathered still preliminary evidence supporting its antipsychotic (AP) efficacy, while highlighting its advantageous side effect profile and good tolerability. Targeting similar pathways, PEA may be considered as a viable alternative to CBD with implications for many therapeutic areas, due to its established safety and the development of formulations maximizing its bioavailability (33, 37).

Overall, the present review demonstrated that PEA may be involved in different psychotic phenotypes. Also, it found initial evidence that PEA levels may reflect the severity of the disorder as well as the stage of illness. Evidence was obtained from both interventional studies addressing the AP potential of PEA supplementation, and observational studies of PEA tone in peripheral blood and in the central nervous system (CNS) in the context of clinical high-risk (CHR) for psychosis and psychotic disorders at different stages of illness.

Some important findings from this systematic review deserve to be highlighted. First, despite its promise, evidence regarding the therapeutic potential of PEA supplementation for psychosis is still limited (51–53) and provides findings about selective efficacy on specific symptoms. In particular, PEA add-on to conventional psychotropic medications did not significantly reduce positive psychotic symptoms (51–53), while ameliorating negative psychotic (52) and acute manic (53) symptoms. Importantly, while not being the focus of this review, results presented here are inconclusive about a potential role of PEA in ameliorating depressive symptoms among psychosis patients (53). Also, in only one study participants were clearly asked to avoid forms of cognitive behavioral therapies during the trial period (52), thus requiring future interventional studies to clearly rule out a potential masking effect of add-on psychotherapy over symptoms.

Second, a line of research investigating endocannabinoids (eCBs) tone in blood among subjects with genetic vulnerability to psychosis (i.e., unaffected twins of SCZ patients) (46), CHR individuals (47), untreated first-episode psychosis (FEP) patients (49), and longer-course SCZ patients (46, 48), converged on the evidence of increased PEA plasma levels as compared to healthy subjects. Also, among CHR patients, the more severe the clinical picture (i.e., greater symptom severity) and the risk profile (i.e., more severe childhood trauma), the higher were the PEA levels (47). Based on PEA-related neurobiological mechanisms, the finding of augmented PEA release across different populations may reflect an endogenous attempt to restore homodynamic balance under disease conditions (28, 29). However, follow-up studies revealed that PEA plasma levels are no longer heightened in AP-treated SCZ patients after 0.6 years of treatment, and further decreased at 5.1 years from baseline (49), potentially suggesting that such biological self-regulation of PEA levels is lost as the diseases progresses. Interestingly, a 2- to 4-week CBD treatment among SCZ patients was associated with higher PEA levels (44) when compared with a 2- to 4-week AP treatment (44), possibly indicating a CBD-specific property in sustaining PEA tone.

Further, differently from SCZ spectrum disorders, PEA plasma level increase was less pronounced in bipolar disorder (BPAD) patients and unaffected twins of BPAD patients, when compared to healthy subjects (46), perhaps accounting for existing phenotypical discrepancies within the affective psychosis group. In fact, PEA plasma levels were greater among BPAD patients having first episode as depression and increased consistently as a function of the lifetime number of depressive episodes (50). Instead, the occurrence of positive psychotic symptoms, manic symptoms, and hypomanic episodes, was correlated with reduced PEA tone (50). Based on evidence that BPAD patients with manic onset have an older age at diagnosis and a longer duration of untreated illness than those with depressive onset (58), and that polarity of episodes over time often reflects polarity at onset (59), such different findings among BPAD patients may suggest a changing pattern in PEA levels over time. In other words, PEA plasma levels would be higher in the first phases of the disorder, while declining because of disease progression, in line with what observed for non-affective psychosis, and thus strengthening the rationale for its supplementation.

Third, CNS PEA levels were found to be augmented in the cerebrospinal fluid (CSF) of young adults with SCZ and schizophreniform disorder (43), whereas reduced in postmortem cerebellar samples of AP-free middle-aged SCZ patients (45), as compared to healthy controls. Similarly, brain PEA levels did not differ between AP-treated middle-aged SCZ patients and healthy controls (45). Again, it can be assumed that PEA levels are elevated in the early stages of psychosis, potentially reflecting an innate compensatory mechanism, before dropping concomitantly to disease progression. With reference to AP treatment, while on one hand it did not appear to increase PEA levels, on the other it is not clear if it prevented a more pronounced longer-term decrease (45).

Only two studies assessed PEA levels in the prefrontal cortex (PFC) of preclinical models of psychosis. They did not show any significant differences as compared to the control condition (54, 55). Further animal studies will need to yield a more robust understanding of the neurobiological mechanisms involving PEA in psychosis.

The findings of this review should be seen considering some strengths and limitations. Despite supporting PEA tone alterations in psychosis and effectiveness of PEA supplementation as a therapeutic strategy, research in this field needs to be expanded, especially to fully comprehend the potential of PEA supplementation as add-on therapy for psychosis across all its symptoms dimensions. Indeed, evidence points toward a beneficial effect of PEA over negative psychotic symptoms and acute manic symptoms, reasonably due to the protective role of the compound against altered neuroinflammation and related mechanisms (28, 29), while PEA effect over positive psychotic symptoms remains to be clarified. Further, future follow-up studies will have to investigate whether PEA effect is sustained in the longer-term. Besides, to date, evidence of PEA effect as monotherapy in the clinical stages preceding full-blown psychosis onset to prevent the risk of progression is totally lacking and is worth of exploration. Finally, PEA levels appear to be altered in psychosis. However, further clarification is needed as to whether PEA tone is altered to a different extent depending on the stage of illness and whether this can be considered a biomarker of psychosis. In line with this, whether any AP treatment may be beneficial through PEA tone modulation requires additional investigation. Biobehavioral comparisons between CBD and PEA are also worth of consideration, especially whether CBD antipsychotic effects are mediated by PEA signaling and similar effects can be obtained directly supplementing PEA. The latter would be inevitably more advantageous, due to its safer profile and shorter biological distance from the therapeutic target.

## 5. Conclusions

This systematic review provided a first overview of all observational and experimental studies of PEA and its biobehavioral correlates in psychosis. Although in its infancy and still limited, research in this field is primarily carried out on humans and provides evidence for both alterations in PEA signaling, implications for psychosis-related behavioral features, and benefits from PEA supplementation. In particular, PEA may be useful to improve negative psychotic symptoms and manic symptoms. Noteworthy, no serious adverse events were reported across all human studies investigating its administration, further supporting PEA potential effectiveness and elevated safety as a therapeutic intervention in psychosis.

## Author contributions

Conceptualization, methodology, validation, resources, writing—review and editing, and visualization: RB, FP, AC, SB, MB, and MC. Software, data curation, writing—original draft preparation, supervision, and project administration: RB and MC. Investigation: RB, MB, and MC. All authors have read and agreed to the published version of the manuscript.
